# AI misuse of retracted literature: A comparative study of ChatGPT4o, deepseek, and grok 3 in stem cell research

**DOI:** 10.1007/s00114-025-02036-5

**Published:** 2025-11-03

**Authors:** Lan Yao, Tianshu Gu, Xuexin Li, Yan Jiao, Minghui Li, J. Carolyn Graff, Yulan Li, Weikuan Gu

**Affiliations:** 1 College of Health Management, Harbin Medical University, Heilongjiang. 157 Baojian Road, Harbin, 150081 Heilongjiang China; 2 Department of Orthopedic Surgery and BME-Campbell Clinic, University of Tennessee Health Science Centre, Memphis, TN 38163 USA; 3 Department of Clinical Pharmacy and Translational Science, University of Tennessee Health Science Center, Memphis, TN 38103 USA; 4 Department of Biostatistics, Vanderbilt University, 2525 West End Ave. Ste 1100, Nashville, TN 37203 USA; 5 The Second Affiliated Hospital of Harbin Medical University, Harbin, China; 6 College of Nursing, University of Tennessee Health Science Center, Memphis, TN 38163 USA; 7 Lt. Col. Luke Weathers, Jr. VA Medical Center, 116 N Pauline St., Memphis, TN 38105 USA; 8 Department of Pharmaceutical Sciences, University of Tennessee Health Science Center, Memphis, TN 38163 USA

**Keywords:** AI, Article, Cell, ChatGPT, DeepSeek, Grok3, Publication, Retraction, Stem

## Abstract

DeepSeek and Grok 3 appear as strong competitors to AI models, particularly the widely accepted model, ChatGPT. The accuracy of the utilization of data in retracted scientific articles has proven to be a significant challenge for AI as an assistant in scientific research. It is critical to understand whether and how three AI models handle information from retracted articles when they answer scientific questions. We collected retracted articles and used AI models to generate questions and analyzed the answers. The answers were compared and evaluated among three AI models. Here we show that these three models utilized 84 out of 93 retracted articles in their answers about stem cells. ChatGPT4o retrieved 74 out of 93 (80%) articles and recognized the retract status for 46 (62%) of them. DeepSeek only found one retracted article and did not realize its retraction status. Grok 4 retrieved 69 (74%) articles and recognized the retraction status of 46 (67%) of them. In cases when the retracted articles were not identified, ChatGPT fabricated articles 5 times out of 19 (26%) for its answers. Grok 3 fabricated 15 articles out of 24 (63%) for its answers. In 82 times of 93 (88%) answers, DeepSeek fabricated the articles in various forms. The answering styles from ChatGPT4o, DeepSeek, and Grok 3 are characterized by accurate and straightforward, a tangential structure and guesswork, and comprehensive and detailed answers, respectively. Analysis with non-retracted articles revealed the similar patterns of these models. This study suggests that, while no model is perfect, DeepSeek performed the worst when facing in-depth scientific real-world challenges. Much improvement has to be made before any of these AI models become problem-free and valuable for scientists.

**Supplementary Information:**

The online version contains supplementary material available at 10.1007/s00114-025-02036-5.

## Introduction

Recently, driven by breakthroughs in deep learning, increasing computational power, and the availability of massive datasets, the field of AI technology has experienced significant growth (Mandl 2025). One example is that PathChat produced more accurate and pathologist-preferable responses to diverse queries related to pathology (Lu et al. 2024). Notably, DeepSeek has just emerged as a formidable challenger in the AI landscape, particularly with its focus on open-weight large language models (LLMs) optimized for efficiency and scalability (Dreyer 2025; Gibney 2025a; Peng et al. 2025). With superior reasoning capabilities, multilingual proficiency, and cost-effective deployment while maintaining transparency, DeepSeek has become a serious competitor in the AI field for public use and a variety of applications. While DeepSeek brings surprising challenges to other AI models (Gibney 2025b, 2025c; Conroy and Mallapaty 2025; Zeng et al. 2025), Grok 3, which combines superior reasoning with extensive pretraining knowledge, was released on February 18, 2025 (Butts, n.d.; Grok 3 Beta 2025). It claims to have leading performance across both academic benchmarks and real-world user preferences.

AI fabrication refers to the use of AI (especially generative AI) to produce synthetic, often deceptive content—such as text, images, audio, or video—that mimics real-world information but is artificially generated. Generative AI tools: Models like GPT (text) generate content from prompts, often indistinguishable from human-created or real content. The content may be designed to mislead (e.g., fake news) or used harmlessly, but its "fabricated" nature lies in being artificially constructed rather than capturing reality. Although both DeepSeek and Grok 3 have attracted considerable attention from the public, their capabilities in the application of medical research have not been deeply explored. The potential values and limitations of the existing early models, such as ChatGPT, have been reported. The limitations for medical science include data bias and generalizability, inability to easily integrate into real-world clinical workflows, and a heavy reliance on clean, labeled, and high-quality medical data, but not timely updates with literature. As such, most early AI models lack the utilization of updated scientific publications to produce research hypotheses and recommendations for disease treatment. In particular, previous studies found that ChatGPT fabricates data and literature (Hua et al. 2023; Li et al. 2024). While it seems to be improving itself and avoiding fabricating articles, it is still facing difficulty in recognizing the untrustworthy publications, such as the retracted articles (Gu et al. 2025).

Retracting problematic articles helps to maintain the accuracy and integrity of the biomedical literature. However, spreading misinformation about retracted articles by AI models harms the medical research as well as the clinical practice (Bengio et al. 2024). DeepSeek and Grok 3 pose a challenge to other models, they may not work well on the scientific literature identification, as they appear recently and may not have learned enough on the selection of the source of a specific literature or lack the access to original resources (Liu 2025; Smith 2025). Our goal is to present a real challenge to both of them by examining how retracted articles in stem cells are treated by Grok 3 and DeepSeek, in comparison with ChatGPT, a well-recognized existing model. We assume that DeepSeek and Grok3 lack the capacity in medical literature searching and recognition of retracted articles, while ChatGPT may have already learned to a certain degree to distinguish between retracted and non-retracted articles.

## Materials and methods

### AI models and procedures

**Fig. 1:**
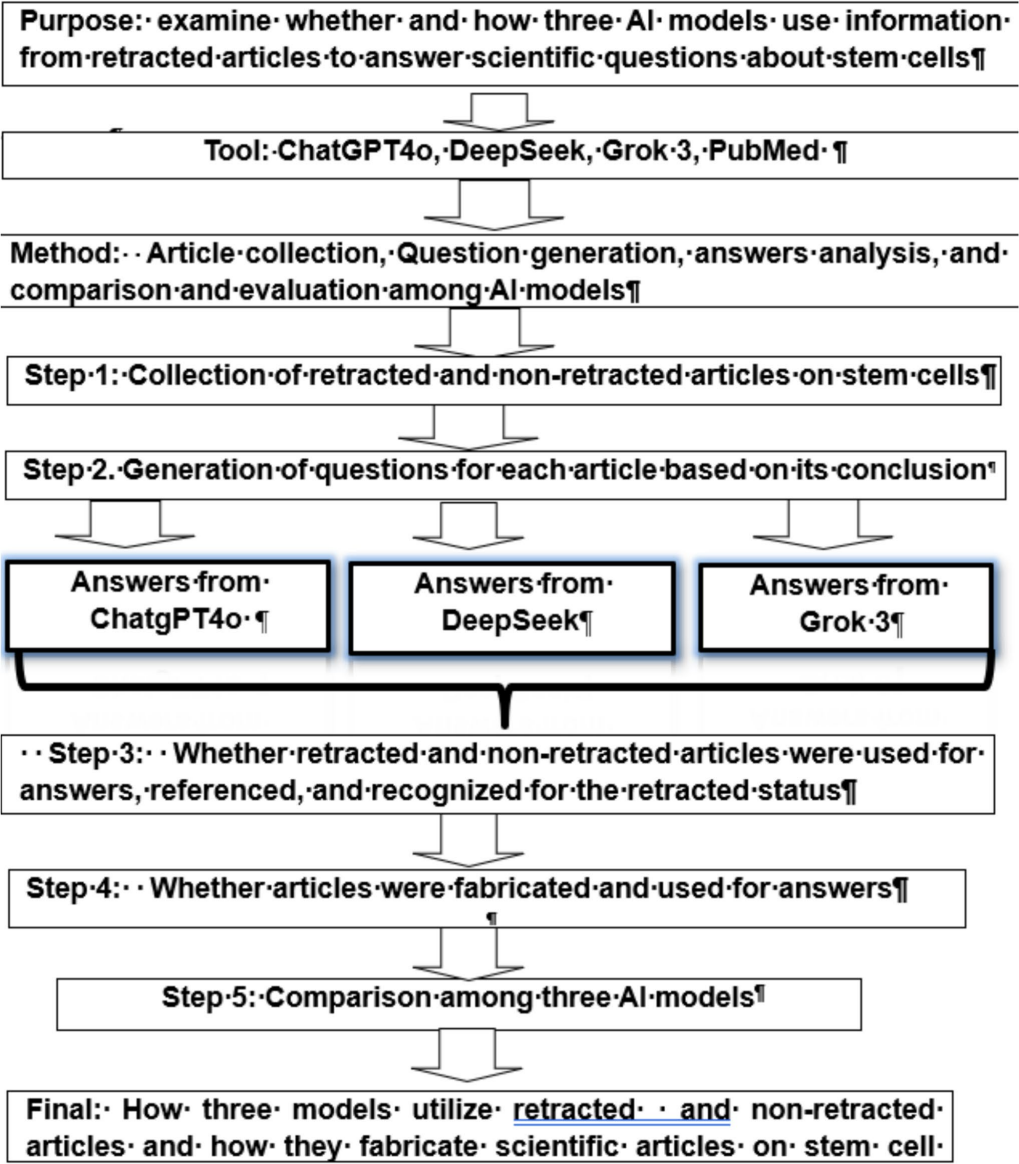
Procedure to investigate whether and how AI models answer questions based on retracted and non-retracted articles in stem cell research. The top three columns provide the purpose, tools and methods for the study. Following by the 5 steps of the analysis. In step 1, the retracted articles on the stem cells are collected. In step 2, the questions are generated based on the retracted article and were used to ask three AI models. In step 3, we determine whether the retracted articles were utilized to answer questions. In step 4, in case the retracted articles were utilized, we exam whether the information of the articles was fabricated or not. Step 5 is the comparison of the utilization of retracted articles among the three AI models. Finally, the utilization of retracted articles by three AI models were summarized

The currently updated version of the three AI models, ChatGPT4o, DeepSeek-v3, and Grok 3, were used for this hypothesis testing. The test procedure is illustrated in Fig. 1.

### *Article collection*

A literature search was performed in PubMed between December 3, 2024 and February 24, 2025to identify English-language articles published but retracted, using two sets of keywords “retraction [Title] stem cell [Title]” and “retracted [Title] stem cell [Title]”. Information on articles was collected in a word document for data analysis. Only retracted research articles were included for the study. Other types of articles, such as reviews and clinical trials, were eliminated from the collection.

### Question development for AI models

Questions were developed based on the conclusion of the articles for every retracted article. For example, if the conclusion of an article is “human bone marrow stem cell (hBMSC)-derived exosomes overexpressing miR-34a may be instrumental for the therapeutic targeting and clinical management of glioblastoma multiforme (GBM)”, the question is “Which study concludes that hBMSC-derived exosomes overexpressing miR-34a may be instrumental for the therapeutic targeting and clinical management of GBM?” Every conclusion-based question was used to ask these three AI models (Supplementary Material). Since the questions were developed specifically for the retracted articles, it is expected that a good model could retrieve the most retracted articles, if not all, when answering the questions.

### Comparison among three AI models

The answers provided by three models were collected and compared. Four key questions were used to compare the answers from three AI models:

- Whether the answer is based on the retracted article,
- Whether the retracted article was cited as the reference to support the answers,
- Whether the models fabricate or legitimate misattribution or paraphrase the references to support their answers,
- Whether the AI model is aware of the retracted publication.

Finally, the answers were analyzed and summarized.

### Test with non-retraced articles

We conducted a test with 20 non-retracted articles. The 20 research articles were published in the same year and the same journals as the first 20 retracted articles. The first non-retracted research article was chosen for the study when searching for articles using journal names and date of the publication of the retracted articles. The questions of 20 articles were asked the three models in two sets, each with10 questions. The analysis of answers to the question was the same as for the retracted articles.

### Statistical analysis

Values of number of retracted and cited articles are calculated as percentage, counts, and 95% Wilson CI whenever it is applicable. The lengths of answers were summarized as means of number of words and Standard deviation (SD).

## Results

Eventually, in a collaborative effort, 96 retracted articles were identified with the keywords “retraction [Title] stem cell [Title]” and 37 retracted articles were identified with the keywords "retracted [Title] stem cell [Title]”. For this study, we selected 93 research articles from the total of 133 identified articles for the test. For each of these 93 articles, one question was asked of all three models (See Supplementary Table 1). Although both DeepSeek and Grok3 are open to the public, they have time delays after answering a certain number of questions, while ChatGPT does not. No model indicated unable to answer any questions.

## Issue of answers based on the retracted articles

**Fig. 2:**
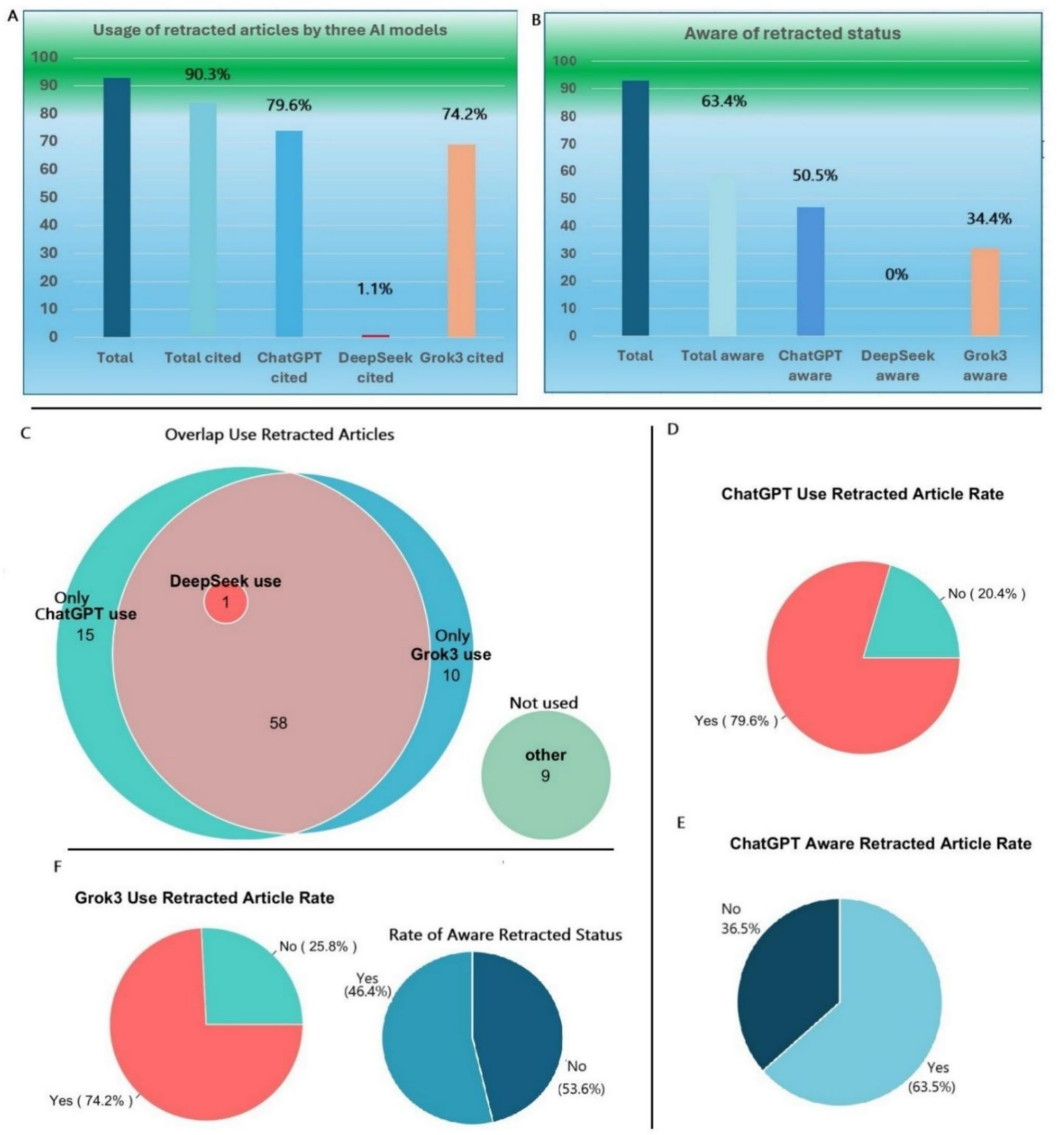
The number of retracted articles utilized by AI models and overlapped article usage among three models. 2A: Usage of retracted articles by three AI models. 2B: Rate of awareness of the retracted articles by three AI models. 2C: Overlap in usage of retracted articles by three AI models. 2D: Rate of usage of retracted articles by ChatGPT. 2E: Rate of awareness of retracted articles by ChatGPT. 2F: Rate of usage of retracted articles and awareness of retraction status by Grok 3

ChatGPT demonstrated the best capability in identifying the specific article based on the content of the question (Table 1). The majority of the answers, 74 out of 93 times, from ChatGPT were based on retracted articles (Fig. 2). One of these 74 answers was not based on the retracted article in question but on a different retracted article. In 53 instances of answers, Grok 3 answers were based on the retracted articles. In only one instance, the answer from DeepSeek was based on the retracted article. The utilization of retracted articles as bases for their answers among these three models showed significant differences. The same article used in one model does not necessarily mean the other model also used it. Among 74 retracted articles that ChatGPT answers questions were based on, DeepSeek answered the questions based on one of these retracted articles, and 59 were used by Grok 3. Among those 69 times that Grok 3 used retracted articles for questions, ChatGPT answered the question with retracted articles in 59 times, while DeepSeek did not use any.

**Table 1:** Summary of AI model performance on retracted articles

Model	Used Retracted	Recognized Retraction	Fabricated References	Answer Length
ChatGPT	79.6% (74/93) [70.3–86.5]	49.5% (46/93) [39.5–59.4]	5.4% (5/93) [2.3–12.0]	177.0 ± 57.2 (n = 93)
DeepSeek	1.1% (1/93) [0.2–5.8]	0.0% (0/93) [0.0–4.0]	88.2% (82/93) [80.0–93.3]	180.2 ± 62.5 (n = 92)
Grok 3	74.2% (69/93) [64.5–82.0]	34.4% (32/93) [25.5–44.5]	16.1% (15/93) [10.0–24.9]	299.7 ± 149.4 (n = 93)

## The capability of recognition of the retracted status of articles

ChatGPT demonstrated its best capability in recognizing retracted status among these three models. Among the 93 answers, ChatGPT used the retracted articles as references 74 times. However, in 47 times, it pointed out the retraction status of the articles. In each case, ChatGPT explained the reason for retraction and advised users that data from the study should be interpreted with caution. DeepSeek used the one retracted article as a reference for its answer. It did not state the retraction status of the retracted article. In 32 out of the 69 times, when Grok 3 used the retracted articles as references for its answers, it realized that the articles have been retracted and mentions the reason of the retraction.

## Mode of answers among three models

**Fig. 3:**
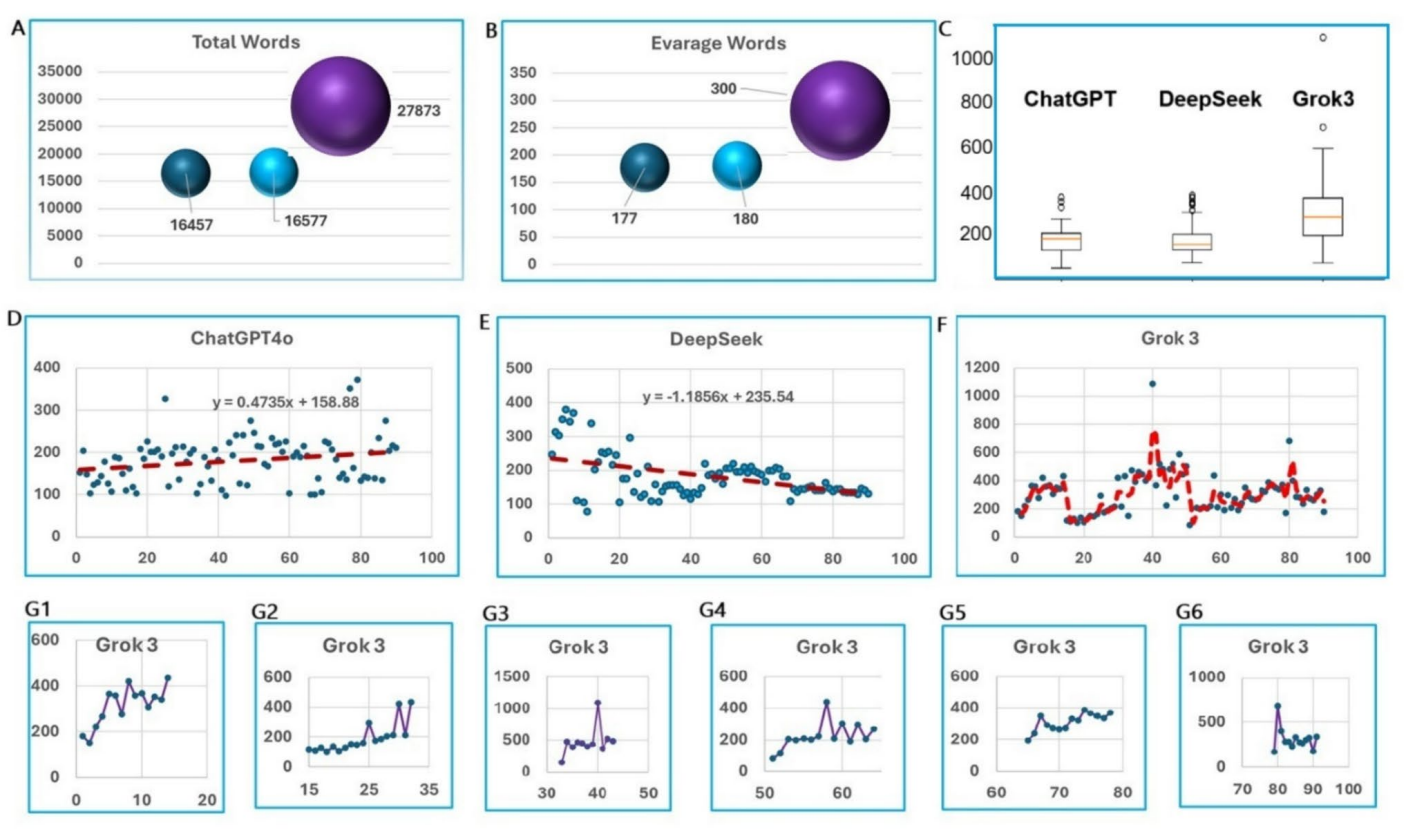
Number of words used for answers by three models and their patterns of learning. 3A: Total number of words used for answers by three models. 3B: Average number of words used for answers by three models. 3C. Number of words and standard deviation used for every answer by three models. 3D to 3Fs, The X- axis is the article numbers while the Y-axis is the number of words in each number of articles. Regress lines in 3D and 3E show the trend of the sizes of answers along the sequential of the questions. 3D: Patterns of answers over time by ChatGPT. 3E: Patterns of answers over time by DeepSeek. 3F: Patterns of answers over time by Grok 3. F1-F6: Patterns of answers in each interval by Grok 3 during test

The answers from each model have their distinct patterns. These differences are reflected in the length of the answers, the order and structure of items in answering the questions (Fig. 3).

Interestingly, we noticed that there are differences in the total number of words of answers among these three models. The averages word number is 176 per answer from ChatGPT, 185 from DeepSeek, and 298 from Grok 3. While there is no correlation among the three models, ChatGPT and DeepSeek did not show a significant difference, with a P value of 0.44 by chi-square analysis. However, there are significant differences between ChatGPT and Grok3, and DeepSeek and Grok 3, with p-values of 0.00 and 0.00 respectively.

ChatGPT answers the question with a target on the topic and directly goes to the key compounds of the question and the articles. Its answers generally emphasize the precision and follow a structured pattern including restating the question, searching for relevant studies, and then providing detailed information about the studies. The format of the answers is consistent with all similar patterns except the first one, which is the simplest (Fig. 3A).

For the DeepSeek, it follows a tangential structured format, which answers questions indirectly and includes 1) A brief statement confirming the existence of the study on the topic and summarizing its key findings; 2) Detailed bullet points explaining mechanisms, pathways, and results; 3) biological or molecular mechanisms behind the phenomenon; 4) the significance of findings, potential applications, and relevance to therapy or disease management; and 5) suggested keywords for further study retrieval in databases like PubMed or Google Scholar or a reference if it is cited. These items answered components related to the question but not directly to the key point of the question. The patterns follow a learning curve, beginning with much information around the answers and then later a more focused similar patterns of fabricating data (Fig. 3B).

The answers from Grok 3 generally with big sizes and thorough contents follow a consistent format: 1). Confirmation of whether a study exists; 2) literature citation (author, journal, year, title); 3). Detailed findings with explanations of key experimental methods, results, and conclusions; 4). Mechanistic Insight (if applicable); And 5). Additional information, including the mechanisms involved and the retraction status, whenever it is realized. However, while the overall pattern of responses remains structured and informative, the similar learning patterns repeat when the questions and answers section restart after a pause (Fig. 3C, D, E, F, F1 to F6). Throughout every period from start to end, when the questions continue, there is increasing complexity in the level of detail provided, including regarding the credibility of sources, particularly regarding retractions, thus, a critical evaluation, rather than simple reporting. In this way, Grok 3 demonstrated a short memory in the learning curve, and it learns from every time when starts.

## Issue of fabricating reference

**Fig. 4:**
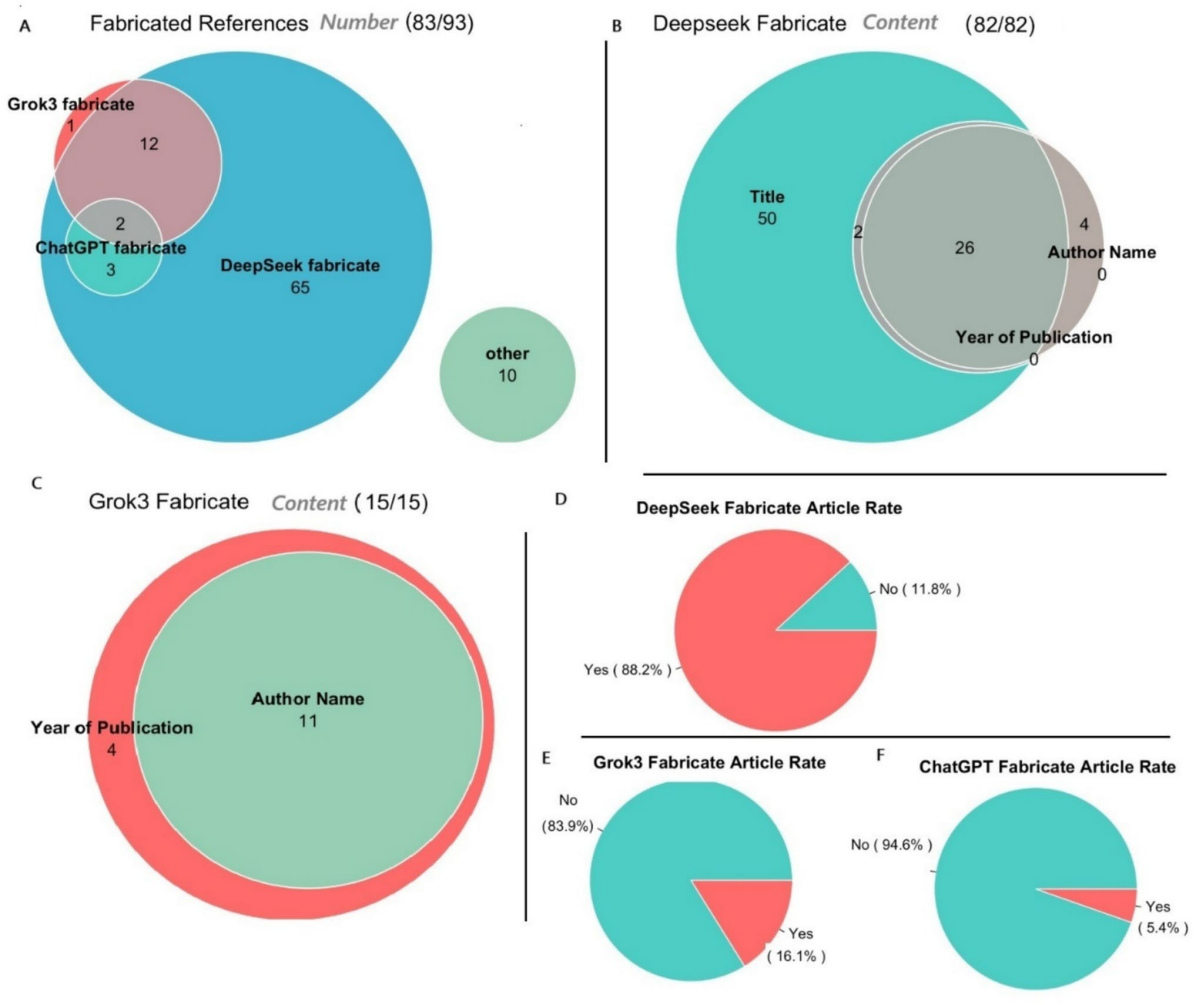
Data on fabricating articles by three AI models. 4A: Total number of fabricated articles and the overlapping among the three models. 4B: Components of fabrication among fabricated articles by DeepSeek. 4C: Components of fabrication among fabricated articles by Grok 3. 4D,4D, 4F: Rate of fabricating articles among three AI models

According to our AI answers, all three models fabricated the references, but the numbers and style of fabrication differ. Our investigation indicated that ChatGPT significantly improved its ability to avoid fabricating articles as references (Supplementary Table 2). For all the answers to the 93 questions, ChatGPT fabricated articles 5 times for its answers (Fig. 4). In 4 cases, ChatGPT provided a fake title of the publication without the journal or year of the publication (Supplementary Material). In the other case, it provided the journal and year of publication without a title. The fabricated titles of the articles are either an incomplete version of existing articles or a similar title to existing articles (Supplementary Material).

Grok 3 fabricated 15 articles for its answers. In contrast to ChatGPT, the 11 fabricated references by Grok 3 contain not only the title, but also the years of publications, names of authors (last name of the first author), and the journal names (Supplementary Table 3). For the rest of the 4 fabricated articles, it provided the fabricated title, journal, and year of publication, except for the names of authors (first author). In addition, Grok 3 also provided a detailed explanation of the content of the fabricated article (Supplementary Material). Again, it reflects its style of robustness in answering questions.

On the other hand, DeepSeek revealed its significant problem in fabricating data. In 82 of the 93 answers, DeepSeek fabricated the articles in a variety of forms (Supplementary Table 4). The 11 times that DeepSeek did not fabricate the article included when answering the first 7 questions one additional time, when it described the findings without giving specific information about the publication. and in one time it utilized the retracted article for its answers and in 2 times it provided a non-retracted article. For the remaining 82 times, it fabricates articles in several forms. The first set of the article fabrication mainly provides a fake title of the article. Usually, it started with: 1) “The study/you are referring to is likely titled (Title of fabricated article)”; 2). “The study that suggests/demonstrated (conclusion of study in question), is likely: (title of fabricated article)” and 3) “The study you are referring to is likely titled: (Title of fabricated article)” (Supplementary Material).

Later, when answering more than half of the questions, DeepSeek provided fabricated articles with more information, similar to that of Grok 3. “One such/possible/relevant study that aligns with this description is: (Title of the fabricated article) or similar titles”.In such a case, the title of an article that later on, the pattern was changed to “The study that (topic in question), is: (title of the fabricated article) by (fabricated last name of the first author) et al., published in (Fabricated name of the journal) (fabricated year of publication)”. In most cases, we could not find it by either searching the PMC or Google. In some cases, the search with the fabricated title retrieved the retracted article.

## Issue in the utilization of non-retracted articles

**Fig. 5:**
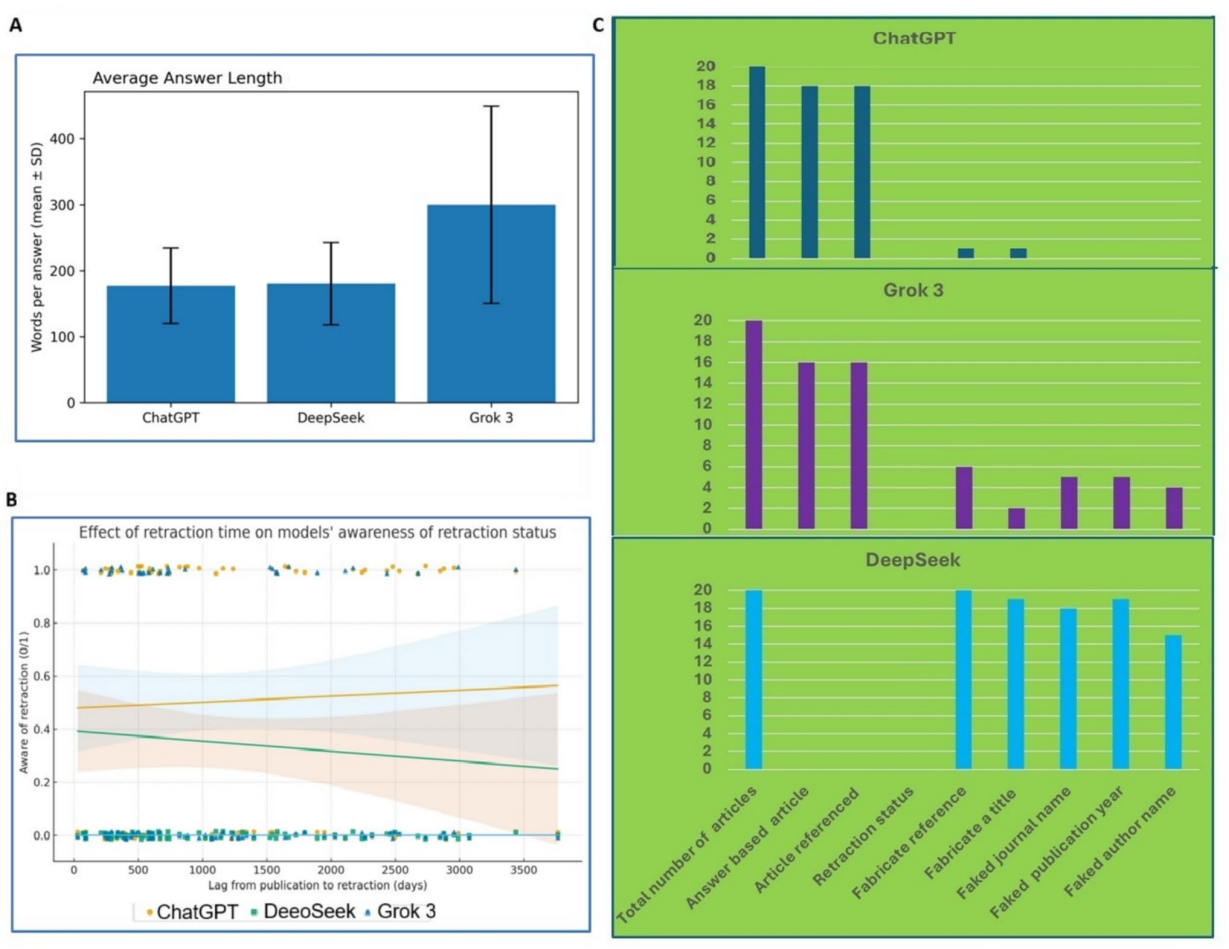
Data of usage of non-retracted articles by three AI models. **A**. The sizes of answers to questions by three AI models. **B**. Different components and patterns of answers of three AI models. **C**. Different components of answers of three models

None of three models regard any non-retracted article as the retracted article. However, the answers among these models showed a significant difference, similar to the questions of retracted articles (Supplementary Table 5–7). For the first ten questions, ChatGPT correctly identified 8 articles for its answers. Among the two cases that ChatGPT did not identify the articles, in one time, ChatGPT provided an altered title for the targeted article (Fig. 5). In the other case, ChatGPT described the content without mentioning the title of the article. When answering the second set of the questions, ChatGPT described the contents and provided links to every article that directly relevant to the question. In contract, DeepSeek did not correctly identify any of the articles in the two set of the questions. Instead, it fabricated articles in the answers to all 20 questions. In 19 cases, it provided faked article titles and publication years. In 18 cases, it provided faked names of journals, and in 15 times, it provided fabricated names of the authors. The answers from Grok 3 showed the complexities of its behaviors. It provided the correct titles of the 16 articles and used them as the sources for answers. Among the 4 cases that it did not identify the title of the articles, in two times, it fabricated the titles of the articles alone with other publication information. In one time, it fabricated publication journal and year without title and author names. In another case, it did not provide any information of the articles but described the contents of the article. Furthermore, in three cases when it identified the correct tiles of the articles, it provided the wrong other information such as name of the journal, publication year and/or name of the authors.

## Discussion

There are significant differences in the precision of identifying articles that answer the questions among three AI models. The questions are developed based on the abstracts of the articles; theoretically, all three models will be capable of accessing the contents of the abstracts of the articles. However, in most cases, ChatGPT was more capable of finding the retracted articles, answering the questions based on the articles, and reminding users about the retracted status of the articles. While in almost all cases, DeepSeek did not seem to realize that the articles and the answers were not based on the articles. In many cases, Grok 3 was able to identify the retracted articles, but randomly notifies the retracted status. We believe that these three models represent the current level of AI development. Our data indicated that recognizing the retracted articles presents a challenge, possibly to all AI models.

Fabrication of data remains a problem for all AI models. We observe a significant improvement in resisting fabricated references by ChatGPT, while it is not yet completely error-free. On the other hand, DeepSeek, as a new model, has a long way to go to overcome this weakness. Grok 3, although it performed a little better than DeepSeek, also needs a considerable amount of effort to face the challenge. In terms of style, Grok 3 and the latter half of the questions from DeepSeek seem designed to give a perfect answer to the questions from users. However, in the case of the fabricated articles, Grok 3 consistently fabricates plausible references by giving seemingly all the compounds of the faked article to make it appear so real. The fabrication process for articles differs between the Grok 3 and DeepSeek. Grok 3 consistently fabricates “perfect references” when fabricating articles almost every time, while DeepSeek appears to learn step by step to fabricate “perfect reference” when fabricating articles.

As we expected, none of the AI models mistakenly regarded non-retracted articles as the retracted article. However, our investigation reveals significant problem of DeepSeek in fabricating articles. In all 20 cases, DeepSeek did not able to correctly recognize any publication information in any article. Rather, it fabricated the publication information in all cases. In most cases, it fabricated not only the title but also the journals, years and authors of the publications. Grok 3 identified the correct title of the majority of the articles while fabricated information of the publications in 6 cases. The pattens of the answers are similar to the answers to the retracted articles. Answers from ChatGPT is precise and short, while answers from DeepSeek and Grok 3 are long and complete, even in the case of fabricating the citations.

Overall, ChatGPT performed the best. It identified the retracted article the most, recognized the retraction status the most, and fabricated references the least. DeepSeek performed the worst among these three models. In most cases, it could not identify the retracted articles. In case it finds the article, it does not recognize the retraction status at all. Whenever it does not find the retracted article, it fabricates articles for its answer.

It is known that there are differences in model architecture, training data, or retrieval-based methods between ChatGPT and the other two, DeepSeek and Grok (Wu et al. 2025; Agarwal et al. 2025). However, it is not clear whether any of these differences is the cause of such a difference in the utilization of the retracted articles. The differences in the number of parameters, model style and process power may affect the speed and capacity of the analyses, but it is not necessary to have a direct connection with the organization of retracted articles. Another significant difference is that ChatGPT was produced earlier than the other AI model. Its problem in fabrication of data was reported as early as in 2023 (Grok 3 Beta 2025) when both DeepSeek and Grok3 were not in public. It is possible that ChatGPT leant and trained to improve its utilization in literature. Further study by expertise in AI technology may reveal the real cause of these differences.

We suggest that the designers of these AI models integrate the retracted articles as part of the future training process. Because a retract marker was posted on the face page of almost all of the retracted articles, training for the recognition of the retracted markers by AI models may enable these models to recognize the difference between the non-retracted and retraction status of the articles and caution of the readers whenever it is necessary.

The time frame of the retraction and the publication of the articles in our study spreads into several decades. However, for the AI models, it mainly the capacity in recognition of the markers of retraction on the face page of the articles. This is more related to AI design and training than the time of retraction of the articles. As long as the AI is capable of reading the article page, whether it mentions the status of the retraction will most likely depends on its capabilities in recognition of the retraction markers and their training to alarm users for the retraction status. Thus, the time frame of the retraction of the articles in our study may not directly correlate with the capability of recognition of the retraction of the articles by AI.

As for the limitations, we only used articles in the study of stem cells, which may be too specific for the new models such as DeepSeek or Grok 3. The performance of these models in other less specific fields or areas, such as public health, may be different. We only asked one question and did not train or remind the model for a correct answer. With additional training, it is possible that the new models of DeepSeek or Grok 3 may improve quickly.

## Conclusion

In summary, currently, no perfect AI models exist that can be used for the biomedical literature search and knowledge seeking. Users must take great caution when using these models for scientific articles and content. Using retracted articles to answer scientific questions by AI model harms scientific integrity as it perpetuates known errors, misinforms future research, and can lead to significant clinical consequences in medicine. Although our study used articles from only study of stem cells and may not represent the situation from all of the rest of the research field, it is important for AI models to take necessary measures to improve the output of the models and avoid misleading users.

## Supplementary Information

Below is the link to the electronic supplementary material.

Supplementary file1 (DOCX 283 KB)

Supplementary file2 (DOCX 42 KB)

Supplementary file3 (DOCX 24 KB)

Supplementary file4 (DOCX 29 KB)

Supplementary file5 (DOCX 29 KB)

Supplementary file6 (DOCX 19 KB)

Supplementary file7 (DOCX 19 KB)

Supplementary file8 (DOCX 19 KB)

## Data Availability

All data are included in the manuscript and the supplementary materials.
