# Supplementary material for: AI misuse of retracted literature: A comparative study of ChatGPT4o, deepseek, and grok 3 in stem cell research
Source: Naturwissenschaften. 2025 Nov 3;112(6):85. doi: 10.1007/s00114-025-02036-5 (PMC12583397; doi:10.1007/s00114-025-02036-5)
Supplement: Supplementary file 2 — Supplementary file2 (DOCX 42 KB) [file 114_2025_2036_MOESM2_ESM.docx]

Supplementary Table 1. Basic information on utilization of retracted articles by three AI models.

| **Retracted Article #** | **Retracted Article Title** | **Original Paper/Date** | **Retraction or Other Notices** | **Was the answer from ChatGPT based on the retracted article?** | **The article referenced or not by ChatGPT** | **Was the answer from Deep Seek based on the retracted article?** | **The article referenced or not by Deep Seek** | **Was the answer from Grok 3 based on the retracted article?** | **The article referenced or not by Grok 3** |
| --- | --- | --- | --- | --- | --- | --- | --- | --- | --- |
| 1 | Osteocyte-derived exosomes induced by mechanical strain promote human periodontal ligament stem cell proliferation and osteogenic differentiation via the miR-181b-5p/PTEN/AKT signaling pathway | 7/17/2020 | 11/5/2024 | Yes | Yes | No | No | Yes | Yes |
| 2 | PHGDH Expression Is Required for Mitochondrial Redox Homeostasis, Breast Cancer Stem Cell Maintenance, and Lung Metastasis | 8/1/2016 | 9/4/2024 | Yes | Yes | No | No | Yes | Yes |
| 3 | G-Protein-Signaling Modulator 2 Expression and Role in a CD133+ Pancreatic Cancer Stem Cell Subset | 1/23/2019 | 8/14/2024 | Yes | Yes | No | No | Yes | Yes |
| 4 | Effect of resveratrol and mesenchymal stem cell monotherapy and combined treatment in management of osteoporosis in ovariectomized rats: Role of SIRT1/FOXO3a and Wnt/β-catenin pathways | 5/30/2021 | 10/7/2024 | Yes | Yes | No | No | Yes | Yes |
| 5 | Spermatogonial stem-cell-derived neural-like cell transplantation enhances the functional recovery of a rat spinal cord injury model: characterization of evoked potentials | 10/16/2023 | 6/11/2024 | No | No | No | No | Yes | Yes |
| 6 | Intraperitoneal Injection of Graphene Oxide Nanoparticle Accelerates Stem Cell Therapy Effects on Acute Kidney Injury | 2/11/2020 | 6/11/2024 | Yes | Yes | No | No | Yes | Yes |
| 7 | Platelet-derived growth factor receptor-α and -β promote cancer stem cell phenotypes in sarcomas | 6/19/2018 | 5/29/2024 | Yes | Yes | No | No | Yes | Yes |
| 8 | A predictive model of herpes zoster after allogeneic hematopoietic stem cell transplantation: VZV reactivation following antiviral prophylaxis discontinuation | 7/9/2023 | 4/26/2024 | Yes | Yes | No | No | No | No |
| 9 | Transfer of microRNA-221 from mesenchymal stem cell-derived extracellular vesicles inhibits atherosclerotic plaque formation | 12/2/2020 | 6/2/2024 | No | No | No | No | Yes | Yes |
| 10 | Knockdown of zinc finger protein 267 suppresses diffuse large B-cell lymphoma progression, metastasis, and cancer stem cell properties | 1/8/2022 | 12/15/2024 | Yes | Yes | No | No | Yes | Yes |
| 11 | N-Cadherin Promotes Recruitment and Migration of Neural Progenitor Cells from the SVZ Neural Stem Cell Niche into Demyelinated Lesions | 7/16/2014 | 12/13/2023 | Yes | Yes | No | No | Yes | Yes |
| 12 | Overexpression of cortistatin alleviates oxygen/glucose-deprivation-induced ER stress and prompts neural stem cell proliferation via SSTR2 | 4/11/2020 | 12/13/2023 | No | No | No | No | No | No |
| 13 | Protective effect of mesenchymal stem cell-conditioned medium on hepatic cell apoptosis after acute liver injury | 10/15/2023 | 11/8/2024 | Yes | Yes | No | No | No | No |
| 14 | LncRNA MALAT1 increases the stemness of gastric cancer cells via enhancing SOX2 mRNA stability | 7/6/2023 | 8/13/2024 | Yes | Yes | No | No | No | No |
| 15 | OTUB2 Facilitates Tumorigenesis of Gastric Cancer Through Promoting KDM1A-Mediated Stem Cell-Like Properties | 9/27/2021 | 5/23/2023 | Yes | Yes | No | No | Yes | Yes |
| 16 | miR-381 Regulates Neural Stem Cell Proliferation and Differentiation via Regulating Hes1 Expression | 10/2/2015 | 3/29/2023 | Yes | Yes | No | No | Yes | Yes |
| 17 | Generation of a homozygous ARHGAP11B knockout hiPSC line by CRISPR/Cas9 system | 5/6/2022 | 4/6/2023 | Yes | No | No | No | No | No |
| 18 | Mesenchymal stem cell-derived exosome miR-542-3p suppresses inflammation and prevents cerebral infarction | 1/6/2021 | 1/16/2023 | Yes | Yes | No | No | Yes | Yes |
| 19 | Bone Marrow Mesenchymal Stem Cell-Derived Exosomal MicroRNA-133a Restrains Myocardial Fibrosis and Epithelial-Mesenchymal Transition in Viral Myocarditis Rats Through Suppressing MAML1 | 7/2/2021 | 12/9/2022 | Yes | Yes | No | No | Yes | Yes |
| 20 | MicroRNA-15b shuttled by bone marrow mesenchymal stem cell-derived extracellular vesicles binds to WWP1 and promotes osteogenic differentiation | 11/16/2020 | 11/10/2022 | Yes | Yes | No | No | Yes | Yes |
| 21 | Mesenchymal stem cell-derived exosomes exert ameliorative effects in type 2 diabetes by improving hepatic glucose and lipid metabolism via enhancing autophagy | 6/8/2020 | 10/20/2022 | Yes | Yes | No | No | Yes | Yes |
| 22 | Mesenchymal stem cell-derived exosomal miR-146a reverses diabetic β-cell dedifferentiation |  |  | No | No | No | No | Yes | Yes |
| 23 | Bone Marrow Mesenchymal Stem Cell-Derived Exosomal MicroRNA-126-3p Inhibits Pancreatic Cancer Development by Targeting ADAM9 | 7/7/2019 | 8/22/2022 | No | No | No | No | No | No |
| 24 | GREM2 maintains stem cell-like phenotypes in gastric cancer cells by regulating the JNK signaling pathway | 10/18/2019 | 12/21/2022 | Yes | Yes | No | No | Yes | No |
| 25 | Secreted Frizzled-Related Protein 4 Inhibits Glioma Stem-Like Cells by Reversing Epithelial to Mesenchymal Transition, Inducing Apoptosis and Decreasing Cancer Stem Cell Properties | 6/1/2015 | 5/5/2022 | Yes | Yes | No | No | Yes | Yes |
| 26 | Chemokine receptor 7 overexpression promotes mesenchymal stem cell migration and proliferation via secreting Chemokine ligand 12 | 1/9/2018 | 3/15/2022 | Yes | No | No | No | Yes | Yes |
| 27 | Long non-coding RNA SNHG3, induced by IL-6/STAT3 transactivation, promotes stem cell-like properties of gastric cancer cells by regulating the miR-3619-5p/ARL2 axis | 2/4/2021 | 2/4/2022 | Yes | Yes | No | No | Yes | Yes |
| 28 | Ang II-AT2R increases mesenchymal stem cell migration by signaling through the FAK and RhoA/Cdc42 pathways in vitro | 7/12/2017 | 11/8/2024 | Yes | Yes | No | No | Yes | Yes |
| 29 | LINC00337 Regulates KLF5 and Maintains Stem-Cell Like Traits of Cervical Cancer Cells by Modulating miR-145 | 8/14/2020 | 12/30/2021 | Yes | Yes | No | No | Yes | Yes |
| 30 | Radiation-induced glucocorticoid receptor promotes CD44 + prostate cancer stem cell growth through activation of SGK1-Wnt/β-catenin signaling | 8/9/2019 | 1/10/2022 | Yes | Yes | No | No | Yes | Yes |
| 31 | Rbm46 regulates mouse embryonic stem cell differentiation by targeting β-Catenin mRNA for degradation | 2/17/2017 | 11/9/2021 | Yes | Yes | No | No | No | No |
| 32 | KIAA1429 promotes osteosarcoma progression by promoting stem cell properties and is regulated by miR-143-3p | 5/19/2020 | 12/20/2021 | Yes | Yes | No | No | Yes | Yes |
| 33 | Human bone marrow-derived mesenchymal stem cell-secreted exosomes overexpressing microRNA-34a ameliorate glioblastoma development via down-regulating MYCN | 12/4/2019 | 10/4/2021 | Yes | Yes | Yes | Yes | Yes | Yes |
| 34 | Transplantation of Mouse Induced Pluripotent Stem Cell-Derived Podocytes in a Mouse Model of Membranous Nephropathy Attenuates Proteinuria | 10/29/2019 | 7/5/2021 | Yes | Yes | No | No | Yes | Yes |
| 35 | Antibacterial and osteogenic stem cell differentiation properties of photoinduced TiO2 nanoparticle-decorated TiO2 nanotubes | 10/5/2015 | 7/16/2021 | Yes | Yes | No | No | Yes | Yes |
| 36 | LncRNA ANRIL Regulates Ovarian Cancer Progression and Tumor Stem Cell-Like Characteristics via miR-324-5p/Ran Axis | 1/19/2021 | 3/30/2021 | Yes | Yes | No | No | Yes | Yes |
| 37 | Neural stem cell conditioned medium alleviates Aβ25-35 damage to SH-SY5Y cells through the PCMT1/MST1 pathway | 7/19/2020 | 10/16/2020 | No | No | No | No | Yes | Yes |
| 38 | Role of miR-132/methyl-CpG-binding protein 2 in the regulation of neural stem cell differentiation | 2/16/2021 | 5/16/2021 | Yes | Yes | No | No | Yes | Yes |
| 39 | Bone mesenchymal stem cell-derived exosomal microRNA-29b-3p prevents hypoxic-ischemic injury in rat brain byactivating the PTEN-mediated Akt signaling pathway | 2/3/2020 | 11/23/2020 | Yes | Yes | No | No | Yes | Yes |
| 40 | IL-1β-induced, matrix metalloproteinase-3-regulated proliferation of embryonic stem cell-derived odontoblastic cells is mediated by the Wnt5 signaling pathway | 10/15/2014 | 12/1/2020 | Yes | Yes | No | No | Yes | Yes |
| 41 | Bone morphogenetic protein-induced cell differentiation involves Atg7 and Wnt16 sequentially in human stem cell-derived osteoblastic cells | 9/10/2016 | 12/1/2020 | Yes | Yes | No | No | Yes | Yes |
| 42 | Cytokines induce MMP-3-regulated proliferation of embryonic stem cell-derived odontoblast-like cells | 7/20/2014 | 11/8/2024 | Yes | Yes | No | No | No | No |
| 43 | Differentiation of human iPSCs into VSMCs and generation of VSMC-derived calcifying vascular cells | 8/31/2018 | 5/4/2020 | No | No | No | No | No | No |
| 44 | Endothelial/Mesenchymal Stem Cell Crosstalk Within Bioprinted Cocultures | 3/26/2020 | 6/26/2020 | No | No | No | No | Yes | Yes |
| 45 | Identification and Characterization of Cells with Cancer Stem Cell Properties in Human Primary Lung Cancer Cell Lines | 8/3/2013 | 4/29/2020 | Yes | Yes | No | No | Yes | Yes |
| 46 | Targeted suicide gene therapy for glioma using human embryonic stem cell-derived neural stem cells genetically modified by baculoviral vectors | 2/19/2012 | 4/27/2020 | Yes | Yes | No | No | Yes | Yes |
| 47 | Interleukin-1β-Induced Autophagy-Related Gene 5 Regulates Proliferation of Embryonic Stem Cell-Derived Odontoblastic Cells | 4/20/2015 | 10/18/2019 | Yes | Yes | No | No | Yes | Yes |
| 48 | Glioma Gene Therapy Using Induced Pluripotent Stem Cell Derived Neural Stem Cell | 10/3/2011 | 9/3/2019 | No | No | No | No | No | No |
| 49 | Brain-Tumor-Regenerating 3D Scaffold-Based Primary Xenograft Models for Glioma Stem Cell Targeted Drug Screening | 1/14/2019 | 8/12/2019 | Yes | Yes | No | No | Yes | Yes |
| 50 | Endocrine disruptors induce perturbations in endoplasmic reticulum and mitochondria of human pluripotent stem cell derivatives | 8/9/2017 | 2/2/2019 | Yes | Yes | No | No | Yes | Yes |
| 51 | Human Cardiac Stem Cell Differentiation Is Regulated by a Mircrine Mechanism | 3/29/2011 | 2/12/2019 | Yes | Yes | No | No | Yes | Yes |
| 52 | The Ephrin A1-EphA2 System Promotes Cardiac Stem Cell Migration After Infarction | 4/29/2011 | 2/15/2019 | Yes | Yes | No | No | Yes | Yes |
| 53 | Notch-1 induces Epithelial-mesenchymal transition consistent with cancer stem cell phenotype in pancreatic cancer cells | 8/1/2011 | 6/1/2018 | Yes | Yes | No | No | Yes | Yes |
| 54 | Effect of umbilical cord mesenchymal stem cell in peri-implant bone defect after immediate implant: an experiment study in beagle dogs | 10/15/2014 | 8/15/2016 | Yes | Yes | No | No | Yes | Yes |
| 55 | Therapeutic Effects of Umbilical Cord Blood Derived Mesenchymal Stem Cell-Conditioned Medium on Pulmonary Arterial Hypertension in Rats | 11/4/2015 | 7/5/2016 | Yes | Yes | No | No | Yes | Yes |
| 56 | Activated K-Ras and INK4a/Arf Deficiency Promote Aggressiveness of Pancreatic Cancer by Induction of EMT Consistent With Cancer Stem Cell Phenotype | 3/22/2013 | 10/23/2016 | No | No | No | No | No | No |
| 57 | Over-expression of FoxM1 leads to epithelial-mesenchymal transition and cancer stem cell phenotype in pancreatic cancer cells | 9/11/2011 | 8/11/2016 | Yes | Yes | No | No | No | No |
| 58 | Tanshinone IIA Pretreatment Renders Free Flaps against Hypoxic Injury through Activating Wnt Signaling and Upregulating Stem Cell-Related Biomarkers | 10/9/2014 | 5/20/2016 | Yes | Yes | No | No | No | No |
| 59 | The noncoding RNA mistral activates Hoxa6 and Hoxa7 expression and stem cell differentiation by recruiting MLL1 to chromatin | 9/16/2011 | 2/5/2015 | Yes | Yes | No | No | Yes | Yes |
| 60 | IFATS collection: selenium induces improvement of stem cell behaviors in human adipose-tissue stromal cells via SAPK/JNK and stemness acting signals | 10/26/2008 | 12/31/2013 | No | No | No | No | Yes | Yes |
| 61 | The stem cell factor antibody enhances the chemotherapeutic effect of adriamycin on chemoresistant breast cancer cells | 5/29/2012 | 10/22/2013 | Yes | Yes | No | No | Yes | Yes |
| 62 | Sp100 as a potent tumor suppressor: accelerated senescence and rapid malignant transformation of human fibroblasts through modulation of an embryonic stem cell program | 12/1/2010 | 8/1/2013 | Yes | Yes | No | No | No | No |
| 63 | characterizing the clinical relevance of an embryonic stem cell phenotype in lung adenocarcinoma | 12/15/2009 | 3/15/2012 | Yes | Yes | No | No | Yes | Yes |
| 64 | High glucose via NOX-dependent ROS generation and AKT activity promotes adipose-derived stem cell de-differentiation | 1/24/2012 | 4/10/2012 | Yes | Yes | No | No | No | No |
| 65 | Systemic signals regulate ageing and rejuvenation of blood stem cell niches | 1/28/2010 | 10/14/2010 | No | No | No | No | Yes | Yes |
| 66 | Spontaneous human adult stem cell transformation | 4/15/2005 | 8/15/2010 | No | No | No | No | No | No |
| 67 | Ex vivo expansion of hematopoietic stem cell by fusion protein TAT-Zfx | 2/13/2009 | 10/16/2009 | Yes | Yes | No | No | No | No |
| 68 | High-dose myeloablative radioimmunotherapy of mantle cell non-hodgkin lymphoma with the iodine-131-labeled chimeric anti-CD20 antibody C2B8 and autologous stem cell support: results of a pilot study. | 2/15/2002 | 12/15/2005 | No | No | No | No | Yes | Yes |
| 69 | Tripartite motif 16 suppresses breast cancer stem cell properties through regulation of Gli‑1 degradation via the ubiquitin‑proteasome pathway | 2/3/2016 | 7/5/2024 | Yes | Yes | No | No | Yes | Yes |
| 70 | Fbxw7 and Skp2 Regulate Stem Cell Switch between Quiescence and Mitotic Division in Lung Adenocarcinoma | 8/25/2019 | 3/20/2024 | Yes | Yes | No | No | Yes | Yes |
| 71 | Long non‑coding RNA MALAT‑1 contributes to maintenance of stem cell‑like phenotypes in breast cancer cells | 12/8/2017 | 2/19/2024 | Yes | Yes | No | No | Yes | Yes |
| 72 | Adipose-Derived Mesenchymal Stem Cell-Derived Extracellular Vesicles Rescue Tendon Injury in Rat via the miR-19 a/IGFBP3 Axis | 9/12/2022 | 1/24/2024 | Yes | Yes | No | No | Yes | Yes |
| 73 | Block Copolymer Nanomicelle-Encapsulated Curcumin Attenuates Cerebral Ischemia Injury and Affects Stem Cell Marker Expression by Inhibiting lncRNA GAS5 | 2/17/2023 | 1/24/2024 | Yes | Yes | No | No | No | No |
| 74 | Antioxidative Capacity of Liver- and Adipose-Derived Mesenchymal Stem Cell-Conditioned Media and Their Applicability in Treatment of Type 2 Diabetic Rats | 2/2/2021 | 1/9/2024 | No | No | No | No | No | No |
| 75 | Investigating the Lung Adenocarcinoma Stem Cell Biomarker Expressions Using Machine Learning Approaches | 9/24/2022 | 1/9/2024 | No | No | No | No | No | No |
| 76 | Resetting Proteostasis of CIRBP with ISRIB Suppresses Neural Stem Cell Apoptosis under Hypoxic Exposure | 9/30/2022 | 12/29/2023 | Yes | Yes | No | No | No | No |
| 77 | Human Umbilical Cord Mesenchymal Stem Cell-Derived Exosome Repairs Endometrial Epithelial Cells Injury Induced by Hypoxia via Regulating miR-663a/CDKN2A Axis | 10/12/2022 | 12/29/2023 | Yes | Yes | No | No | Yes | Yes |
| 78 | Mild Hypothermia Promotes Ischemic Tolerance and Survival of Neural Stem Cell Grafts by Enhancing Global SUMOylation | 5/27/2022 | 12/29/2023 | Yes | Yes | No | No | Yes | Yes |
| 79 | Effect of Autologous Stem Cell Transplantation Combined with Modified VTD Regimen on Elderly Patients with Multiple Myeloma and Its Influence on miRNA Cytokines | 2/16/2022 | 12/6/2023 | Yes | Yes | No | No | Yes | Yes |
| 80 | MicroRNA-424-5p Alleviates Isoflurane Anesthesia-Induced Neurotoxicity in Human Embryonic Stem Cell-Derived Neurons by Targeting FASN | 7/13/2022 | 7/26/2023 | Yes | Yes | No | No | No | No |
| 81 | Prognostic Value of Stem Cell Index-Related Characteristics in Primary Hepatocellular Carcinoma | 6/8/2022 | 7/19/2023 | No | No | No | No | Yes | Yes |
| 82 | ECM-Mimicking Hydrogels Loaded with Bone Mesenchymal Stem Cell-Derived Exosomes for the Treatment of Cartilage Defects | 11/3/2022 | 7/19/2023 | Yes | Yes | No | No | Yes | Yes |
| 83 | Adipose-Derived Stem Cell Exosomes Inhibit Hypertrophic Scaring Formation by Regulating Th17/Treg Cell Balance | 10/12/2022 | 7/12/2023 | Yes | Yes | No | No | Yes | Yes |
| 84 | Mesenchymal Stem Cell Exosomal miR-146a Mediates the Regulation of the TLR4/MyD88/NF- κ B Signaling Pathway in Inflammation due to Diabetic Retinopathy | 6/18/2022 | 6/28/2023 | Yes | Yes | No | No | Yes | Yes |
| 85 | miR‑218‑5p inhibits the stem cell properties and invasive ability of the A2B5+CD133‑ subgroup of human glioma stem cells | 2/3/2016 | 10/4/2022 | Yes | Yes | No | No | Yes | Yes |
| 86 | The Favorable Effect of Mesenchymal Stem Cell Treatment on the Antioxidant Protective Mechanism in the Corneal Epithelium and Renewal of Corneal Optical Properties Changed after Alkali Burns | 1/5/2016 | 5/11/2022 | No | No | No | No | Yes | Yes |
| 87 | Silencing of cystatin SN abrogates cancer progression and stem cell properties in papillary thyroid carcinoma | 8/11/2021 | 8/13/2023 | Yes | Yes | No | No | Yes | Yes |
| 88 | The Effects of Cytokines in Adipose Stem Cell-Conditioned Medium on the Migration and Proliferation of Skin Fibroblasts In Vitro | 12/15/2013 | 9/28/2020 | Yes | Yes | No | No | No | No |
| 89 | Serum‑free‑medium‑type mesenchymal stem cell culture supernatant exerts a protective effect on A549 lung epithelial cells in acute lung injury induced by H2O2 | 8/17/2018 | 6/4/2019 | Yes | Yes | No | No | Yes | Yes |
| 90 | Combined treatment with extracorporeal shock‑wave therapy and bone marrow mesenchymal stem cell transplantation improves bone repair in a rabbit model of bone nonunion | 1/17/2018 | 10/18/2018 | Yes | Yes | No | No | Yes | Yes |
| 91 | Evidence of a pluripotent human embryonic stem cell line derived from a cloned blastocyst | 2/12/2004 | 3/12/2004 | No | No | No | No | No | No |
| 92 | Bone mesenchymal stem cell-derived exosomal microRNA-7-5p inhibits progression of acute myeloid leukemia by targeting OSBPL11 | 1/10/2022 | 1/18/2025 | Yes | Yes | No | No | Yes | No |
| 93 | Mesenchymal Stem Cell-Derived Extracellular Vesicles Alleviate M1 Microglial Activation in Brain Injury of Mice With Subarachnoid Hemorrhage via microRNA-140-5p Delivery | 4/19/2022 | 2/4/2025 | Yes | Yes | No | No | Yes | No |
