# Supplementary material for: AI misuse of retracted literature: A comparative study of ChatGPT4o, deepseek, and grok 3 in stem cell research
Source: Naturwissenschaften. 2025 Nov 3;112(6):85. doi: 10.1007/s00114-025-02036-5 (PMC12583397; doi:10.1007/s00114-025-02036-5)
Supplement: Supplementary file 3 — Supplementary file3 (DOCX 24 KB) [file 114_2025_2036_MOESM3_ESM.docx]

Supplementary Table 1. Information of article fabrication by ChatGPT.

| Retracted Article # | Did ChatGPT fabricate references | If Yes, did ChatGPT fabricate a title of article | Did ChatGPT provided faked journal name | Did CahtGPT provided faked year of publication | Did ChatGPT provided a faked author name | How many words and characters did ChatGPT use for its answer |
| --- | --- | --- | --- | --- | --- | --- |
| 1 | No |  |  |  |  | 153 |
| 2 | No |  |  |  |  | 204 |
| 3 | No |  |  |  |  | 149 |
| 4 | No |  |  |  |  | 103 |
| 5 | No |  |  |  |  | 124 |
| 6 | No |  |  |  |  | 129 |
| 7 | No |  |  |  |  | 145 |
| 8 | No |  |  |  |  | 178 |
| 9 | No |  |  |  |  | 126 |
| 10 | No |  |  |  |  | 108 |
| 11 | No |  |  |  |  | 189 |
| 12 | No |  |  |  |  | 187 |
| 13 | No |  |  |  |  | 150 |
| 14 | No |  |  |  |  | 110 |
| 15 | No |  |  |  |  | 162 |
| 16 | No |  |  |  |  | 118 |
| 17 | No |  |  |  |  | 103 |
| 18 | No |  |  |  |  | 209 |
| 19 | No |  |  |  |  | 185 |
| 20 | No |  |  |  |  | 226 |
| 21 | No |  |  |  |  | 202 |
| 22 | No |  |  |  |  | 202 |
| 23 | No |  |  |  |  | 207 |
| 24 | No |  |  |  |  | 191 |
| 25 | No |  |  |  |  | 327 |
| 26 | No |  |  |  |  | 120 |
| 27 | No |  |  |  |  | 197 |
| 28 | No |  |  |  |  | 212 |
| 29 | No |  |  |  |  | 136 |
| 30 | No |  |  |  |  | 214 |
| 31 | No |  |  |  |  | 178 |
| 32 | No |  |  |  |  | 197 |
| 33 | No |  |  |  |  | 207 |
| 34 | No |  |  |  |  | 104 |
| 35 | No |  |  |  |  | 125 |
| 36 | No |  |  |  |  | 190 |
| 37 | Yes | No | Yes | Yes | No | 168 |
| 38 | No |  |  |  |  | 134 |
| 39 | No |  |  |  |  | 207 |
| 40 | No |  |  |  |  | 183 |
| 41 | No |  |  |  |  | 111 |
| 42 | No |  |  |  |  | 98 |
| 43 | Yes | Yes | No | No | No | 224 |
| 44 | No |  |  |  |  | 194 |
| 45 | No |  |  |  |  | 241 |
| 46 | No |  |  |  |  | 127 |
| 47 | No |  |  |  |  | 242 |
| 48 | No |  |  |  |  | 123 |
| 49 | No |  |  |  |  | 275 |
| 50 | No |  |  |  |  | 247 |
| 51 | No |  |  |  |  | 215 |
| 52 | No |  |  |  |  | 214 |
| 53 | No |  |  |  |  | 173 |
| 54 | No |  |  |  |  | 168 |
| 55 | No |  |  |  |  | 235 |
| 56 | No |  |  |  |  | 219 |
| 57 | No |  |  |  |  | 222 |
| 58 | No |  |  |  |  | 202 |
| 59 | No |  |  |  |  | 226 |
| 60 | Yes | Yes | No | No | No | 103 |
| 61 | No |  |  |  |  | 191 |
| 62 | No |  |  |  |  | 201 |
| 63 | No |  |  |  |  | 190 |
| 64 | No |  |  |  |  | 215 |
| 65 | No |  |  |  |  | 192 |
| 66 | No |  |  |  |  | 100 |
| 67 | No |  |  |  |  | 100 |
| 68 | No |  |  |  |  | 139 |
| 69 | No |  |  |  |  | 106 |
| 70 | No |  |  |  |  | 226 |
| 71 | No |  |  |  |  | 222 |
| 72 | No |  |  |  |  | 207 |
| 73 | No |  |  |  |  | 184 |
| 74 | Yes | Yes | No | No | No | 140 |
| 75 | No |  |  |  |  | 150 |
| 76 | No |  |  |  |  | 136 |
| 77 | No |  |  |  |  | 352 |
| 78 | No |  |  |  |  | 164 |
| 79 | No |  |  |  |  | 372 |
| 80 | No |  |  |  |  | 134 |
| 81 | No |  |  |  |  | 143 |
| 82 | No |  |  |  |  | 140 |
| 83 | No |  |  |  |  | 200 |
| 84 | No |  |  |  |  | 139 |
| 85 | No |  |  |  |  | 235 |
| 86 | Yes | Yes | No | No | No | 135 |
| 87 | No |  |  |  |  | 275 |
| 88 | No |  |  |  |  | 204 |
| 89 | No |  |  |  |  | 217 |
| 90 | No |  |  |  |  | 211 |
| 91 | No |  |  |  |  | 98 |
| 92 | No | Yes | Yes | Yes |  | 67 |
| 93 | No | Yes | Yes | Yes |  | 54 |
