# Supplementary material for: AI misuse of retracted literature: A comparative study of ChatGPT4o, deepseek, and grok 3 in stem cell research
Source: Naturwissenschaften. 2025 Nov 3;112(6):85. doi: 10.1007/s00114-025-02036-5 (PMC12583397; doi:10.1007/s00114-025-02036-5)
Supplement: Supplementary file 4 — Supplementary file4 (DOCX 29 KB) [file 114_2025_2036_MOESM4_ESM.docx]

Supplementary Table 2. Information of article fabrication by Grok3.

| Retracted Article # | Did Grok 3 fabricate reference | If Yes, did Grok 3 fabricate a title of article | Did Grok 3 provided faked journal name | Did Grok 3 provided faked year of publication | Did Grok 3 provided a faked author name | Word number |
| --- | --- | --- | --- | --- | --- | --- |
| 1 | No |  |  |  |  | 182 |
| 2 | No |  |  |  |  | 150 |
| 3 | No |  |  |  |  | 222 |
| 4 | No |  |  |  |  | 266 |
| 5 | No |  |  |  |  | 365 |
| 6 | No |  |  |  |  | 358 |
| 7 | No |  |  |  |  | 276 |
| 8 | Yes | Yes | Yes | Yes | Yes | 421 |
| 9 | No | - | - | - | - | 359 |
| 10 | No | - | - |  |  | 368 |
| 11 | No | - | - |  |  | 308 |
| 12 | No | - | - | - | - | 353 |
| 13 | No | No | Yes | - | - | 341 |
| 14 | No | No | - | - | - | 435 |
| 15 | No |  |  |  |  | 118 |
| 16 | No |  |  |  |  | 109 |
| 17 | No | - |  |  |  | 129 |
| 18 | No |  |  |  |  | 102 |
| 19 | No |  |  |  |  | 138 |
| 20 | No |  |  |  |  | 106 |
| 21 | No | No | Np | Yes | Yes | 129 |
| 22 | No | - |  |  |  | 152 |
| 23 | Yes | Yes | Yes | Yes | No | 147 |
| 24 | Yes | Yes | Yes | Yes | Yes | 161 |
| 25 | No | - |  |  |  | 293 |
| 26 | No | - |  |  |  | 173 |
| 27 | No | - |  |  |  | 186 |
| 28 | No | - |  |  |  | 204 |
| 29 | No | - |  |  |  | 214 |
| 30 | No | - |  |  |  | 422 |
| 31 | Yes | Yes | Yes | Yes | No | 214 |
| 32 | No | - |  |  |  | 432 |
| 33 | No | - |  |  |  | 152 |
| 34 | No | - |  |  |  | 473 |
| 35 | No | - |  |  |  | 392 |
| 36 | No | - |  |  |  | 462 |
| 37 | No | - |  |  |  | 445 |
| 38 | No | - |  |  |  | 400 |
| 39 | No | - |  |  |  | 435 |
| 40 | No | - |  |  |  | 1088 |
| 41 | No | - |  |  |  | 366 |
| 42 | No | - |  |  |  | 519 |
| 43 | Yes | Yes | Yes | Yes | No | 482 |
| 44 | No | - |  |  |  | 225 |
| 45 | No | - |  |  |  | 484 |
| 46 | No | - |  |  |  | 518 |
| 47 | No | - |  |  |  | 282 |
| 48 | No | - |  |  |  | 589 |
| 49 | No |  |  |  |  | 444 |
| 50 | No | - |  |  |  | 501 |
| 51 | No | - |  |  |  | 85 |
| 52 | No | - |  |  |  | 116 |
| 53 | No | - |  |  |  | 207 |
| 54 | No | - |  |  |  | 200 |
| 55 | No | - |  |  |  | 210 |
| 56 | Yes | Yes | Yes | Yes | Yes | 203 |
| 57 | Yes | Yes | Yes | Yes | Yes | 222 |
| 58 | No | - |  |  |  | 438 |
| 59 | No | - |  |  |  | 210 |
| 60 | No | - |  |  |  | 302 |
| 61 | No | - |  |  |  | 191 |
| 62 | Yes | Yes | Yes | Yes | Yes | 296 |
| 63 | No | - |  |  |  | 207 |
| 64 | No | - |  |  |  | 269 |
| 65 | No | - |  |  |  | 192 |
| 66 | Yes | Yes | Yes | Yes | Yes | 240 |
| 67 | Yes | Yes | Yes | Yes | Yes | 351 |
| 68 | No | - |  |  |  | 291 |
| 69 | No | - |  |  |  | 271 |
| 70 | No | - |  |  |  | 266 |
| 71 | No | - |  |  |  | 272 |
| 72 | No | - |  |  |  | 332 |
| 73 | Yes | Yes | Yes | Yes | Yes | 324 |
| 74 | Yes | Yes | Yes | Yes | Yes | 389 |
| 75 | No | - |  |  |  | 368 |
| 76 | Yes | Yes | Yes | Yes | Yes | 352 |
| 77 | No | - |  |  |  | 338 |
| 78 | No | - |  |  |  | 372 |
| 79 | No | - |  |  |  | 169 |
| 80 | No | - |  |  |  | 684 |
| 81 | No | - |  |  |  | 401 |
| 82 | No | - |  |  |  | 284 |
| 83 | No | - |  |  |  | 283 |
| 84 | No | - |  |  |  | 235 |
| 85 | No | - |  |  |  | 333 |
| 86 | No | - |  |  |  | 275 |
| 87 | No | - |  |  |  | 262 |
| 88 | Yes | Yes | Yes | Yes | No | 303 |
| 89 | No | - |  |  |  | 330 |
| 90 | No | - |  |  |  | 178 |
| 91 | Yes | Yes | Yes | Yes | Yes | 342 |
| 92 | No |  |  |  |  | 113 |
| 93 | No |  |  |  |  | 77 |
