# Supplementary material for: AI misuse of retracted literature: A comparative study of ChatGPT4o, deepseek, and grok 3 in stem cell research
Source: Naturwissenschaften. 2025 Nov 3;112(6):85. doi: 10.1007/s00114-025-02036-5 (PMC12583397; doi:10.1007/s00114-025-02036-5)
Supplement: Supplementary file 5 — Supplementary file5 (DOCX 29 KB) [file 114_2025_2036_MOESM5_ESM.docx]

Supplementary Table 3. Information of article fabrication by DeepSeek.

| Retracted Article # | Did Deep Seek fabricate reference | If Yes, did DeepSeek fabricate a title of article | Did DeepSeek provided faked journal name | Did DeepSeek provided faked year of publication | Did DeepSeek provided a faked author name | Word Number |
| --- | --- | --- | --- | --- | --- | --- |
| 1 | No | - | - | - | - | 247 |
| 2 | No | - | - | - | - | 313 |
| 3 | No | - | - | - | - | 304 |
| 4 | No | - | - | - | - | 351 |
| 5 | No | - | - | - | - | 381 |
| 6 | No | - | - | - | - | 344 |
| 7 | No | - | - | - | - | 370 |
| 8 | Yes | Yes | Yes | Yes | Yes | 111 |
| 9 | Yes | Yes | Yes | Yes | Yes |  |
| 10 | Yes | Yes | No | No | No | 105 |
| 11 | Yes | Yes | No | No | No | 78 |
| 12 | Yes | Yes | No | No | No | 339 |
| 13 | Yes | Yes | No | No | No | 202 |
| 14 | Yes | Yes | No | No | No | 225 |
| 15 | Yes | Yes | No | No | No | 254 |
| 16 | Yes | Yes | No | No | No | 251 |
| 17 | No | - | - | - | - | 255 |
| 18 | Yes | Yes | No | No | No | 217 |
| 19 | Yes | Yes | No | No | No | 246 |
| 20 | Yes | Yes | No | No | No | 106 |
| 21 | Yes | Yes | No | No | No | 176 |
| 22 | Yes | Yes | Yes | Yes | Yes | 176 |
| 23 | Yes | Yes | No | No | No | 296 |
| 24 | Yes | Yes | No | No | No | 136 |
| 25 | Yes | Yes | No | No | No | 191 |
| 26 | Yes | Yes | No | No | No | 120 |
| 27 | Yes | No | Yes | Yes | Yes | 130 |
| 28 | Yes | No | Yes | Yes | Yes | 212 |
| 29 | Yes | No | Yes | Yes | Yes | 109 |
| 30 | Yes | No | Yes | Yes | Yes | 159 |
| 31 | Yes | Yes | No | No | No | 107 |
| 32 | Yes | Yes | No | No | No | 138 |
| 33 | No | - | - | - | - | 154 |
| 34 | Yes | Yes | No | No | No | 156 |
| 35 | Yes | Yes | No | No | No | 157 |
| 36 | Yes | Yes | No | No | No | 157 |
| 37 | Yes | Yes | No | No | No | 145 |
| 38 | Yes | Yes | No | No | No | 126 |
| 39 | Yes | Yes | No | No | No | 135 |
| 40 | Yes | Yes | No | No | No | 115 |
| 41 | Yes | Yes | No | No | No | 135 |
| 42 | Yes | Yes | No | No | No | 130 |
| 43 | Yes | Yes | No | No | No | 148 |
| 44 | No | - | - | - | - | 220 |
| 45 | Yes | Yes | No | No | No | 186 |
| 46 | Yes | Yes | No | No | No | 189 |
| 47 | Yes | Yes | No | No | No | 176 |
| 48 | Yes | Yes | No | No | No | 193 |
| 49 | Yes | Yes | No | No | No | 160 |
| 50 | Yes | Yes | No | No | No | 206 |
| 51 | Yes | Yes | No | No | No | 206 |
| 52 | Yes | Yes | No | No | No | 220 |
| 53 | Yes | Yes | No | No | No | 195 |
| 54 | Yes | Yes | No | No | No | 195 |
| 55 | Yes | Yes | No | No | No | 210 |
| 56 | Yes | Yes | No | No | No | 193 |
| 57 | Yes | Yes | No | No | No | 211 |
| 58 | Yes | Yes | No | No | No | 198 |
| 59 | Yes | Yes | No | No | No | 193 |
| 60 | Yes | Yes | No | No | No | 187 |
| 61 | Yes | Yes | No | No | No | 167 |
| 62 | Yes | Yes | No | No | No | 199 |
| 63 | Yes | Yes | No | No | No | 200 |
| 64 | Yes | Yes | No | No | No | 208 |
| 65 | Yes | Yes | No | No | No | 204 |
| 66 | Yes | Yes | No | No | No | 183 |
| 67 | Yes | Yes | No | No | No | 183 |
| 68 | Yes | Yes | Yes | Yes | Yes | 108 |
| 69 | Yes | Yes | Yes | Yes | Yes | 145 |
| 70 | Yes | Yes | Yes | Yes | Yes | 136 |
| 71 | Yes | Yes | Yes | Yes | Yes | 145 |
| 72 | Yes | Yes | Yes | Yes | Yes | 144 |
| 73 | Yes | Yes | Yes | Yes | Yes | 150 |
| 74 | Yes | Yes | Yes | Yes | Yes | 153 |
| 75 | Yes | Yes | Yes | Yes | Yes | 142 |
| 76 | Yes | Yes | Yes | Yes | Yes | 142 |
| 77 | Yes | Yes | Yes | Yes | Yes | 142 |
| 78 | Yes | Yes | Yes | Yes | Yes | 163 |
| 79 | Yes | Yes | Yes | Yes | Yes | 147 |
| 80 | Yes | Yes | Yes | Yes | Yes | 138 |
| 81 | Yes | Yes | Yes | Yes | Yes | 144 |
| 82 | Yes | Yes | Yes | Yes | Yes | 144 |
| 83 | Yes | Yes | Yes | Yes | Yes | 136 |
| 84 | Yes | Yes | Yes | Yes | Yes | 134 |
| 85 | Yes | Yes | Yes | Yes | Yes | 134 |
| 86 | Yes | Yes | Yes | Yes | Yes | 135 |
| 87 | Yes | Yes | Yes | Yes | Yes | 129 |
| 88 | Yes | Yes | Yes | Yes | Yes | 147 |
| 89 | Yes | Yes | Yes | Yes | Yes | 141 |
| 90 | Yes | Yes | Yes | Yes | Yes | 131 |
| 91 | No | No | No | No | No | 211 |
| 92 | Yes | Yes | Yes | Yes | No | 123 |
| 93 | Yes | Yes | yes | Yes | No | 124 |
