# Supplementary material for: AI misuse of retracted literature: A comparative study of ChatGPT4o, deepseek, and grok 3 in stem cell research
Source: Naturwissenschaften. 2025 Nov 3;112(6):85. doi: 10.1007/s00114-025-02036-5 (PMC12583397; doi:10.1007/s00114-025-02036-5)
Supplement: Supplementary file 6 — Supplementary file6 (DOCX 19 KB) [file 114_2025_2036_MOESM6_ESM.docx]

Supplementary Table 4. Data of answers for non retracted articles from ChatGPT.

|  | Was the answer from ChatGPT based on the article? | The article referenced or not by ChatGPT | Did ChatGP Mention the retraction status of the publication | Did ChatGPT fabricate a reference | If Yes, did ChatGPT fabricate a title of the article | Did ChatGPT provide faked journal name | Did ChatGPT provide faked year of publication | Did ChatGPT provide a faked author’s name | How many words and characters did ChatGPT use for its answer |
| --- | --- | --- | --- | --- | --- | --- | --- | --- | --- |
| 1 | Yes | Yes | No | No |  |  |  |  | 104 |
| 2 | Yes | Yes | No | No |  |  |  |  | 86 |
| 3 | No | No | No | No |  |  |  |  | 89 |
| 4 | Yes | Yes | No | Yes | Yes | No | No | No | 142 |
| 5 | Yes | Yes | No | No |  |  |  |  | 67 |
| 6 | No | No | No | No |  |  |  |  | 183 |
| 7 | Yes | Yes | No | No |  |  |  |  | 139 |
| 8 | Yes | Yes | No | No |  |  |  |  | 57 |
| 9 | Yes | Yes | No | No |  |  |  |  | 108 |
| 10 | Yes | Yes | No | No |  |  |  |  | 69 |
| 11 | Yes | Yes | No | No |  |  |  |  | 154 |
| 12 | Yes | Yes | No | No |  |  |  |  | 79 |
| 13 | Yes | Yes | No | No |  |  |  |  | 103 |
| 14 | Yes | Yes | No | No |  |  |  |  | 79 |
| 15 | Yes | Yes | No | No |  |  |  |  | 137 |
| 16 | Yes | Yes | No | No |  |  |  |  | 132 |
| 17 | Yes | Yes | No | No |  |  |  |  | 79 |
| 18 | Yes | Yes | No | No |  |  |  |  | 135 |
| 19 | Yes | Yes | No | No |  |  |  |  | 45 |
| 20 | Yes | Yes | No | No |  |  |  |  | 135 |
| Summary | 18.00 | 18.00 | 0.00 | 1.00 | 1.00 | 0.00 | 0.00 | 0.00 | 2122.00 |
