# Supplementary material for: AI misuse of retracted literature: A comparative study of ChatGPT4o, deepseek, and grok 3 in stem cell research
Source: Naturwissenschaften. 2025 Nov 3;112(6):85. doi: 10.1007/s00114-025-02036-5 (PMC12583397; doi:10.1007/s00114-025-02036-5)
Supplement: Supplementary file 7 — Supplementary file7 (DOCX 19 KB) [file 114_2025_2036_MOESM7_ESM.docx]

Supplementary Table 5. Data of answers for non-retracted articles from DeepSeek.

| Article # | Was the answer from DeepSeek based on the article? | The article referenced or not by Deep Seek | Did Deep seek mention the retraction status of the publication | Did Deep Seek fabricate a reference | If Yes, did DeepSeek fabricate a title for the article | Did DeepSeek provide a faked journal name | Did DeepSeek provide a faked year of publication | Did DeepSeek provide a faked author’s name | How many words and characters did Deep seek to use for its answer |
| --- | --- | --- | --- | --- | --- | --- | --- | --- | --- |
| 1 | No | No | No | Yes | Yes | No | No | No | 183 |
| 2 | No | No | No | Yes | Yes | Yes | Yes | Yes | 184 |
| 3 | No | No | No | Yes | Yes | Yes | Yes | Yes | 165 |
| 4 | No | No | No | Yes | Yes | Yes | Yes | No | 168 |
| 5 | No | No | No | Yes | Yes | Yes | Yes | Yes | 210 |
| 6 | No | No | No | Yes | Yes | Yes | Yes | No | 190 |
| 7 | No | No | No | Yes | Yes | Yes | Yes | No | 228 |
| 8 | No | No | No | Yes | Yes | Yes | Yes | Yes | 238 |
| 9 | No | No | No | Yes | Yes | Yes | Yes | No | 236 |
| 10 | No | No | No | Yes | Yes | Yes | Yes | Yes | 232 |
| 11 | No | No | No | Yes | No | No | Yes | Yes | 272 |
| 12 | No | No | No | Yes | Yes | Yes | Yes | Yes | 148 |
| 13 | No | No | No | Yes | Yes | Yes | Yes | Yes | 224 |
| 14 | No | No | No | Yes | No | Yes | Yes | Yes | 209 |
| 15 | No | No | No | Yes | Yes | Yes | Yes | Yes | 214 |
| 16 | No | No | No | Yes | Yes | Yes | Yes | Yes | 232 |
| 17 | No | No | No | Yes | Yes | Yes | Yes | Yes | 197 |
| 18 | No | No | No | Yes | Yes | Yes | Yes | Yes | 226 |
| 19 | No | No | No | Yes | Yes | Yes | Yes | Yes | 204 |
| 20 | No | No | No | Yes | Yes | Yes | Yes | Yes | 207 |
|  | 0 | 0 | 0 | 20 | 19 | 18 | 19 | 15 | 4167 |
