# Supplementary material for: AI misuse of retracted literature: A comparative study of ChatGPT4o, deepseek, and grok 3 in stem cell research
Source: Naturwissenschaften. 2025 Nov 3;112(6):85. doi: 10.1007/s00114-025-02036-5 (PMC12583397; doi:10.1007/s00114-025-02036-5)
Supplement: Supplementary file 8 — Supplementary file8 (DOCX 19 KB) [file 114_2025_2036_MOESM8_ESM.docx]

Supplementary Table 6. Data of answers for non-retracted articles from Grok 3.

| Article # | Was the answer from Grok 3 based on the article? | Article referenced or not by Grok 3 | Was Grok 3 mentioned the retraction status of the publication | Did Grok 3 fabricate reference | If Yes, did Grok 3 fabricate a title of article | Did Grok 3 provided faked journal name | Did Grok 3 provided faked year of publication | Did Grok 3 provided a faked author name | How many words and characters did Grok 3 use for its answer |
| --- | --- | --- | --- | --- | --- | --- | --- | --- | --- |
| 1 | Yes | Yes | No | No |  |  |  |  | 199 |
| 2 | Yes | Yes | No | No |  |  |  |  | 198 |
| 3 | Yes | Yes | No | No |  |  |  |  | 200 |
| 4 | No | No | No | Yes | No | Yes | Yes | No | 216 |
| 5 | Yes | Yes | No | No |  |  |  |  | 281 |
| 6 | Yes | Yes | No | Yes | No | Yes | Yes | Yes | 230 |
| 7 | No | No | No | No |  |  |  |  | 269 |
| 8 | Yes | Yes | No | No |  |  |  |  | 395 |
| 9 | Yes | Yes | No | Yes | No | Yes | No | Yes | 318 |
| 10 | Yes | Yes | No | No |  |  |  |  | 218 |
| 11 | Yes | Yes | No | No |  |  |  |  | 450 |
| 12 | Yes | Yes | No | No |  |  |  |  | 209 |
| 13 | No | No | No | Yes | Yes | Yes | Year | No | 389 |
| 14 | Yes | Yes | No | No |  |  |  |  | 295 |
| 15 | Yes | Yes | No | No |  |  |  |  | 222 |
| 16 | Yes | Yes | No | No |  |  |  |  | 219 |
| 17 | Yes | Yes | No | Yes | No | No | Yes | Yes | 232 |
| 18 | Yes | Yes | No | No |  |  |  |  | 261 |
| 19 | No | No | No | Yes | Yes | Yes | Yes | Yes | 190 |
| 20 | Yes | Yes | No | No |  |  |  |  | 214 |
|  | 16 | 16 | 0 | 6 | 2 | 5 | 5 | 4 | 5205 |
